# Landscape determinants of pelagic and benthic primary production in northern lakes

**DOI:** 10.1111/gcb.16409

**Published:** 2022-09-15

**Authors:** Isolde Callisto Puts, Jenny Ask, Matthias B. Siewert, Ryan A. Sponseller, Dag O. Hessen, Ann‐Kristin Bergström

**Affiliations:** ^1^ Climate Impacts Research Centre, Department of Ecology and Environmental Science Umeå University Umeå Sweden; ^2^ Department of Biosciences Oslo University Oslo Norway

**Keywords:** autotrophic structuring, carbon fertilization, climate change, CO_2_, DOC, GPP, hydrology, land cover

## Abstract

Global change affects gross primary production (GPP) in benthic and pelagic habitats of northern lakes by influencing catchment characteristics and lake water biogeochemistry. However, how changes in key environmental drivers manifest and impact total (i.e., benthic + pelagic) GPP and the partitioning of total GPP between habitats represented by the benthic share (*autotrophic structuring*) is unclear. Using a dataset from 26 shallow lakes located across Arctic, subarctic, and boreal northern Sweden, we investigate how catchment properties (air temperature, land cover, hydrology) affect lake physico‐chemistry and patterns of total GPP and autotrophic structuring. We find that total GPP was mostly light limited, due to high dissolved organic carbon (DOC) concentrations originating from catchment soils with coniferous vegetation and wetlands, which is further promoted by high catchment runoff. In contrast, autotrophic structuring related mostly to the relative size of the benthic habitat, and was potentially modified by CO_2_ fertilization in the subarctic, resulting in significantly higher total GPP relative to the other biomes. Across Arctic and subarctic sites, DIC and CO_2_ were unrelated to DOC, indicating that external inputs of inorganic carbon can influence lake productivity patterns independent of terrestrial DOC supply. By comparison, DOC and CO_2_ were correlated across boreal lakes, suggesting that DOC mineralization acts as an important CO_2_ source for these sites. Our results underline that GPP as a resource is regulated by landscape properties, and is sensitive to large‐scale global changes (warming, hydrological intensification, recovery of acidification) that promote changes in catchment characteristics and aquatic physico‐chemistry. Our findings aid in predicting global change impacts on autotrophic structuring, and thus community structure and resource use of aquatic consumers in general. Given the similarities of global changes across the Northern hemisphere, our findings are likely relevant for northern lakes globally.

## Graphical abstract

Visualization of landscape determinants of pelagic (light green) and benthic (dark green) gross primary production (GPP) along a gradient in colored dissolved organic carbon (DOC; left to right). This DOC gradient is driven by changes in altitude and climate across Arctic (high altitude), subarctic (mid altitude), and boreal (low altitude) regions of northern Sweden. Along this gradient, lakes become browner, more nutrient rich, warmer, and lower in pH (modifying the DIC pool from ${\mathrm{HCO}}_{3}^{-}$ to CO_2_), resulting in a shift from systems where GPP is benthic driven to those where it is pelagic driven. These changes also underpin a unimodal relationship between total GPP and DOC across the region.

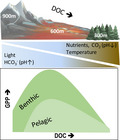

## 1 INTRODUCTION

Global changes such as warming, hydrological intensification, and recovery from acidification have altered seasonality, thermal regimes, and biogeochemistry of northern lakes (Creed et al., 2018; Evans et al., 2005; Laudon et al., 2021). Such changes promote, separately or in combination, higher concentrations of dissolved organic matter (DOM) in lakes, typically referred to as browning or darkening. Elevated DOM inputs to lakes reduce light availability, supplement nutrient supplies, and lead to warmer and more carbon dioxide (CO_2_) supersaturated surface waters, which collectively impacts algal growth (Bartosiewicz et al., 2016; Hessen et al., 2017; Jansson et al., 2012; Jones & Lennon, 2015; Thrane et al., 2014). Most studies of DOM effects in lakes have emphasized changes in gross primary production (GPP) in the water column (pelagic GPP), but effects on autotrophs growing on soft sediments (benthic GPP) are less studied, despite the significant reliance on benthic GPP by higher consumers in lake food webs (Hecky & Hesslein, 1995; Karlsson & Byström, 2005; Vesterinen et al., 2016). As a consequence, it is not settled how global changes in northern landscapes influence the partitioning of GPP between pelagic and benthic habitats, as represented by the benthic share of total GPP (i.e., the *autotrophic structuring*, cf. Higgins et al., 2014), as well as overall lake productivity (benthic + pelagic GPP).

In small and shallow lakes throughout the northern hemisphere, the variance in autotrophic structuring is generally large and influenced by the habitat size, as well as physical (temperature, light), physico‐chemical (nutrients, pH), ecological (grazing, bioturbation), and seasonal processes that mediate the relative amount of GPP by benthic versus pelagic primary producers (Kosten et al., 2009; McCormick et al., 2021; Scheffer, 2004; Schindler & Scheuerell, 2002; Vander Zanden & Vadeboncoeur, 2020). Changes in optical properties and nutrient status of the water are major factors regulating GPP and autotrophic structuring, and drivers of these factors differ across regions (Kosten et al., 2009; Krause‐Jensen et al., 2012). In temperate, nutrient rich landscapes, eutrophic conditions promote pelagic GPP, resulting in increased turbidity and shading of the benthic habitat (Krause‐Jensen et al., 2012; Scheffer & Jeppesen, 2007; Vadeboncoeur et al., 2003). Such lakes can, through complex feedback loops, abruptly shift into clear‐watered states in the presence of macrophytes (Althouse et al., 2014; Kosten et al., 2009; Scheffer, 2004; Schindler & Scheuerell, 2002). However, in northern oligotrophic lakes, shading by colored DOM concentrations, and not by phytoplankton, is the most important inhibiting factor for pelagic and especially benthic algal growth (Ask et al., 2009a; Bergström & Karlsson, 2019; Hansson, 1992; Jones, 1992; Puts et al., 2022; Thrane et al., 2014). Also for such systems, complex feedback loops and sudden changes are expected to occur in response to browning (Spears et al., 2017). Hence, it is implied that in northern lakes, the autotrophic structuring shifts from benthic‐ to pelagic‐dominated systems along gradients of increasing DOM concentrations (Ask et al., 2009a; Rivera Vasconcelos et al., 2018; Vasconcelos et al., 2019).

The general consensus, confirmed by modeling and empirical studies, is that total GPP in northern lakes relates unimodally to DOM concentrations, with an initial increase along a DOM gradient attributed to nutrient supply from DOM, followed by decreased GPP due to light limitation at higher concentrations of DOM (Rivera Vasconcelos et al., 2018; Seekell et al., 2015; Solomon et al., 2015). Yet, unimodal relationships are not always observed, and the global variation in GPP at intermediate DOM concentrations is notably large, indicating that other factors can regulate total lake productivity (Kelly et al., 2018; Seekell et al., 2015). Such factors may also be directly or indirectly related to DOM. For example, photon absorption by DOM and consequent stratification can increase surface water temperatures (Bartosiewicz et al., 2016, 2019; Houser, 2006; Kraemer et al., 2015; Pilla et al., 2018, 2020) and thereby alter pelagic GPP. Similarly, high DOM concentrations can promote CO_2_ supersaturation through biotic and/or abiotic mineralization (Larsen, Andersen, et al., 2011; Nydahl et al., 2019; Sobek et al., 2003). Variation in CO_2_ concentrations can further alter rates of pelagic (Brown et al., 2019; Grasset et al., 2020; Hammer, 2019; Jansson et al., 2012) and possibly benthic GPP (Karlsson et al., 2001), through the so‐called “carbon fertilization” effect, which may be most pronounced at intermediate DOM concentrations with optimal light and nutrient conditions. However, CO_2_ availability is also regulated by the total dissolved inorganic carbon (DIC) concentrations and pH (Hunt et al., 2011), and may be uncoupled from DOM where geogenic inputs of DIC are elevated (Borges et al., 2014; Rantakari & Kortelainen, 2008; Weyhenmeyer et al., 2015). Hence, light availability, temperature, nutrients, and CO_2_ are key drivers of pelagic, benthic, and total GPP that potentially relate to DOM inputs in northern lakes. However, since these drivers may have interactive effects, the net outcome for ecosystem productivity is not straightforward.

The factors that collectively shape autotrophic structuring and total GPP of lakes are, in turn, regulated by catchment and climate features that mediate land–water interactions at regional scales. For example, the concentrations, spectral properties, and nutrient content of DOM in northern lakes differ across the regional landscapes. Specifically, DOM in Arctic and subarctic lakes tends to be lower in concentration, but also less colored and more nutrient poor, when compared to boreal counterparts (Bergström et al., 2020; Isles et al., 2020, 2021; Seekell et al., 2015). Furthermore, the extent of CO_2_ supersaturation (Lapierre et al., 2017; Nydahl et al., 2020; Sobek et al., 2003), total DIC concentrations, and pH (Futter et al., 2014; Weyhenmeyer et al., 2019) can vary substantially in lakes across northern landscapes. This variation reflects climatic factors like air temperatures and hydrology, as well as shifts in land cover (e.g., open areas, wetlands, boreal forests) and connected land‐use characteristics (e.g., afforestation or deforestation) of catchments. At more local scales, lake bathymetry also influences biogeochemical processing and determines the available habitat for GPP. For instance, shallower lakes tend to have intensified epilimnion warming (Bartosiewicz et al., 2019; Kraemer et al., 2015), more benthic habitat relative to the pelagic habitat (Devlin et al., 2016; Godwin et al., 2014), and higher metabolism in general (Staehr et al., 2012).

Here, we present a dataset with autotrophic structuring and total GPP measured during midsummer (June–July) in 26 shallow lakes in three regions, spread across the boreal, subarctic, and Arctic biomes in northern Sweden. We used these data to assess how landscape characteristics, land cover, and hydrology together with bathymetry influence lake biogeochemical characteristics, and evaluate the main drivers and the variability in total GPP and autotrophic structuring among these lakes. Our results illustrate how landscape modifies total GPP and autotrophic structuring through catchment and lake water properties, that impact light and nutrient conditions, and CO_2_ concentrations.

## 2 METHODOLOGY AND APPROACH

### 2.1 Study area, sampling, lake water physico‐chemistry, and bathymetry

**FIGURE 1:**
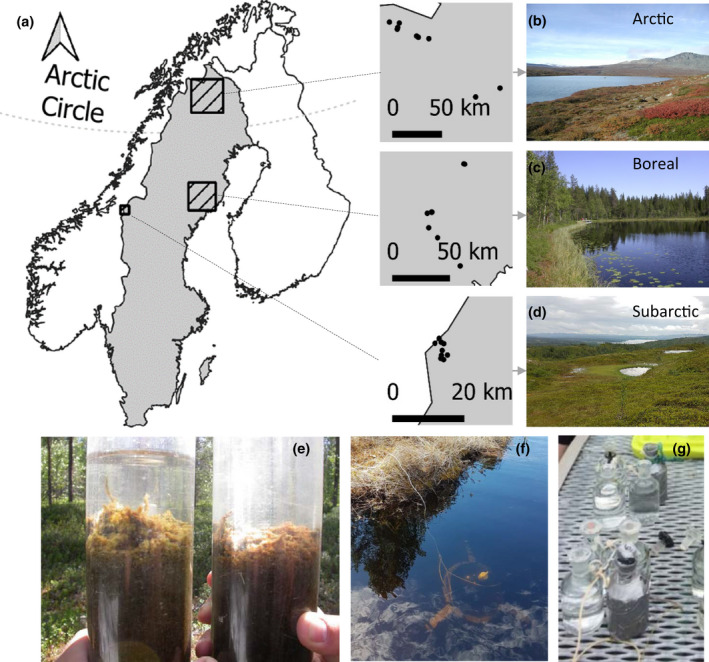
(a) Study locations of the lakes and a picture of the (b) Arctic (Ruozut), (c) boreal (one of the Björntjärn lakes), and (d) subarctic landscape (close to lake Värgtjärnen). Field pictures of methodology for measuring GPP in the (e–f) benthic or (g) pelagic, depicting (e) cores used for the DIC method, and (f) a dome employed for the Dome method, equipped with an oxygen logger, and (g) dark and light bottles with added isotopic tracer that are incubated in the pelagic in situ at different depths.

We compiled data on summer primary productivity in benthic and pelagic habitats sampled between 2005 and 2017, together with water chemistry, from 26 shallow lakes (maximum 3.7–15.8 m deep) from three different sites in northern Sweden, located in the Arctic (Norrbotten), subarctic (Jämtland), and boreal (Västerbotten; Figure 1a) biomes. The data can be found at https://doi.org/10.5061/dryad.vx0k6djvs. Here, we define the biomes (Arctic and subarctic) using the definition of the Arctic Monitoring and Assessment Program based on latitude, elevation, vegetation, and occurrence of permafrost (AMAP, 1998). The three study regions have variable elevation gradients and vegetation cover: in the Artic (270–933 m above sea level [m a.s.l.]), catchments are fed mainly by open areas above the tree line (on average 71%) with occasional deciduous vegetation (8%) and wetlands (7%); subarctic catchments (578–655 m a.s.l.) have approximately equal amounts of open areas (39%) and wetlands (38%); finally, boreal catchments (238–336 m a.s.l.) are dominated by coniferous vegetation (60%) and temporarily deforested areas (12%; Figure 1b–d). The study lakes cover a wide range of dissolved organic carbon (DOC; 1.5–16.3 mg L^−1^) and accompanied total nutrient concentrations (TN: 80.5–501.8 μg L^−1^, TP: 3.9–33.9 μg L^−1^), water color (Kd: 0.3–4.1 m^−1^), and pH (5.3–7.8), DIC (0.4–3.5 mg L^−1^) and consequent CO_2_ concentrations (0.1–1.9 mg L^−1^). The lakes have similar average bathymetry (lake average depth; *z*_avg_), but boreal lakes had higher DOC concentrations and thus darker waters (higher Kd), leading to a lower relative benthic habitat size (%*A*_littoral_; measure combining water color and bathymetry) when compared to the Arctic. The subarctic lakes have similar bathymetry as the other sites, with Kd and %*A*_littoral_ values between Arctic and boreal lake conditions.

Water chemistry, PAR, and temperature were measured on the same dates as GPP measurements. PAR and temperature were measured from the surface to the bottom with 1 m depth intervals at the deepest part of the lake, with additional measurements at 0.25 m and 0.5 m using a handheld probe. Light attenuation coefficients (Kd) of the lake water were calculated as the absolute slope of natural logarithmically transformed photosynthetically active radiation (PAR) against depth. The sum of incoming PAR over the day was retrieved from stations we installed next to the lake. We used the water temperature measured at 0.2 m depth (*T*_water_) as proxy for lake epilimnion temperatures, as most of the GPP takes place at shallow depths, and this is also where internal warming of the epilimnion cause by colored DOC takes place (Pilla et al., 2018). Average air temperatures 1 month before sampling (*T*_air_) were retrieved from weather stations (extracted from https://www.smhi.se) situated closest (within a range of 60 km) to the sampling sites, and we included a temperature decrease of 0.57°C per 100 m elevation difference between station and sampling site (sensu Karlsson et al., 2005 and references therein). Water samples for measuring pH, DOC, DIC, total nitrogen (TN), and phosphorus (TP) were taken at 1 m depth (epilimnion), or in nine cases from composite water samples (unstratified lakes from Ask et al., 2009a). pH was measured directly after sampling and CO_2_ concentrations in the lake water were calculated from DIC, pH, and temperature (https://www.epa.gov for specifics). In brief, DOC was filtered through a 0.45 μm filter (Sarstedt Filtropur), acidified with HCl to an end concentration of 12 mM, and stored in a refrigerator before analyzed. TN and TP (unfiltered) samples were kept frozen until analysis. The DIC concentration was calculated from the headspace CO_2_ concentration in closed vials containing acidified lake water according to Åberg et al. (2007), and were analyzed as soon as possible. More details and specific laboratory operating procedures afterwards can be found in Ask et al. (2009a, 2009b) and Puts et al. (2022) and Appendix S1. Detailed lake bathymetry was acquired through integrated GPS and echo‐sounding depth measurements, from which we calculated lake average depth (*z*_avg_), lake volumes and areas (as a whole, or in different sections), as well as the relative areal size of the littoral benthic habitat (%*A*_littoral_; see Appendix S2: Table S1 for overview parameters).

### 2.2 Gross primary production

GPP was measured between 21 June and 28 July in variable years between 2005 and 2017 in situ in the benthic and pelagic on the same date. Benthic GPP was measured using the Dome method (subarctic) or the DIC method (Arctic and boreal; see Appendix S1 and Puts et al., 2022 for detailed descriptions and comparison). For the DIC method, intact sediment cores with overlaying water were collected in incubation tubes using a gravity corer on three or five depths, and incubated for about 24 h at the depth of collection. GPP rates at the discrete depths were measured by tracking changes in DIC concentrations between the onset and end of the incubation period in sealed off dark (respiration [R]) or transparent (R + GPP) tubes (Figure 1e). In the Dome method, three transparent domes equipped with a miniDOT oxygen logger (MiniDOT website, n.d.) were gently placed on the sediment at a different depth each, and O_2_ metabolism of the separated sediment area was measured for 24 h (Figure 1f). Benthic GPP rates at the three discrete depths were derived from net ecosystem production (NEP) using the R‐package Lake Metabolizer (Winslow et al., 2016) and by assuming that GPP equals NEP plus R (by assuming R is oxygen loss during dark period; 24 h metabolism, more details in Puts et al. [2022] and Appendix S1). Pelagic GPP was measured at the surface, 0.25, 0.5 m, and at following 1 m depth intervals, where the deepest measurement depended on the lake depth and water turbidity. Measurements were done by incubating transparent glass bottles in situ filled with water from the sampling depth, with additional incubations in dark bottles at the most shallow and deepest measurements, for about 4 h around noon using a ^14^C isotopic tracer (sensu Schindler et al., 1972; Figure 1g). The GPP values measured for 4 h midday at varying depths were converted to daily values by relating the midday measurements to the ratio of incident PAR during incubation time in relation to the daily PAR (24 h). We used averages for duplicate or triplicate measurements of pelagic and benthic rates. An average lake GPP (mg C m^−2^ day^−1^) was calculated for the benthic (benthic GPP_lake‐average_) and pelagic (pelagic GPP_lake‐average_) habitat. Benthic and pelagic GPP daily rates at discrete depths were upscaled to a lake average per m^2^ (benthic and pelagic GPP_lake‐average_; mg C m^−2^ day^−1^) by integrating the GPP rates over the corresponding lake surface (benthic) or lake volume (pelagic) per depth interval, and relating the sum to the total lake area. The total average GPP of the lake (total GPP_lake‐average_) is expressed as the sum of benthic and pelagic GPP_lake‐average_ (mg C m^−2^ day^−1^), and autotrophic structuring is expressed as the relative amount (%) of GPP that takes place in the benthic habitat (Appendix S2: Table S1).

### 2.3 Catchment and landscape features

Digital elevation models (DEMs) with 2 × 2 m resolution were downloaded from Lantmäteriet (https://www.lantmateriet.se/sv/geodata/vara‐produkter/produktlista/markhojdmodell‐nedladdning/), and a national inventory land cover classification with 10 m × 10 m resolution (2017–2019) was downloaded from naturvårdsverket (https://www.naturvardsverket.se). The catchment area (*A*_catchment_) for each lake and average topographic wetness indexes of the corresponding catchment (TWIs; Beven & Kirkby, 1979) were calculated in Whitebox GAT (https://www.whiteboxgeo.com), by preprocessing (filling, breaching, and manual corrections based on orthomap Bing) and delineating DEMs, taking into account interfering infrastructures when relevant. TWI is a proxy for the relative wetness of a point based on the topography, with higher values predicting wetter areas (Beven & Kirkby, 1979). Land cover for each watershed was extracted by overlaying the catchment and land cover raster using QGIS (Appendix S2: Figure S1 for maps showing land cover per catchment). Incidental land covers present in only a few catchments and covering <5% were excluded. The remaining land cover types were reclassified into “water”, “wetland”, “open area”, “deciduous forest”, “coniferous forest”, or “temporarily deforested” (Appendix S2: Table S2). Average yearly runoff values (years 1981–2010; *R*_yearly_, mm year^−1^) of the corresponding area for the delineated catchments were downloaded from Vattenweb (https://www.smhi.se/data/hydrologi/vattenwebb). The lake hydraulic retention time (HRT, year^−1^) was estimated by dividing lake (m^3^) by the annual discharge (m^3^ year^−1^), and drainage ratio (DR) was expressed as catchment area divided by lake area (Appendix S2: Table S1 for equations and units).

### 2.4 Statistical analyses

We investigated how catchment characteristics (land cover and hydrology; *R*_yearly_, TWI, HRT, DR) together with bathymetry influence lake biogeochemical characteristics using redundancy analysis (RDA; Canoco v.5.1; ter braak & Šmilauer, 2018). In an RDA with supplementary variables, a set of response variables (water chemistry) is determined by a set of explanatory variables (land cover), which, together with supplementary variables (hydrology) that do not influence the statistics, we summarized in a biplot where the spread of these variables within the dataset can be explored. For this analysis, we included altitude and air temperature (*T*_air_) along with the water chemistry dataset, as altitude (but not latitude) is a major driver of lake DOC in the northern Swedish landscape (Karlsson et al., 2005; Figure 1a), and our dataset covers lakes across a wide range in altitudes. In addition, to identify the main drivers of GPP for further analyses described below, we used multiple linear regression (MLR) with forward selection (FS) for all four GPP measurements (benthic, pelagic, and total GPP_lake‐average_, and autotrophic structuring: see Appendix S2: Table S3).

**FIGURE 2:**
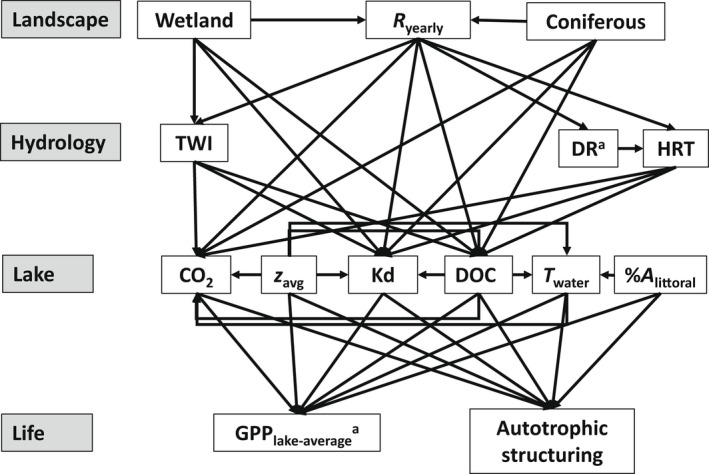
Structural equation model (SEM) used in the path analysis. Arrows indicate the direction of causality. *R*_yearly_, average yearly runoff (mm); TWI, topographic wetness index; DR, drainage ratio; HRT, hydraulic retention time (year^−1^); CO_2_, carbon dioxide in lake water (mg L^−1^); *z*_avg_, average lake depth (m); *T*_water_ = temperature at 0.2 m (°C); %*A*_littoral_, relative areal size of the littoral benthic habitat (%). Variables marked with superscript “a” are log‐transformed.

Finally, we investigated if certain catchment properties shape total GPP_lake‐average_ and autotrophic structuring by use of a path analysis, using the lavaan package (Rosseel, 2012) in R. A path analysis is a type of MLR, where presumed causal relationships between several variables are calculated in standardized path coefficients that can be visually summarized in a structural equation model (SEM) where major influencing variables are statistically selected. In a SEM, presumed causal relationships are predefined using a priori knowledge, and go one way (variables cannot influence each other). We based presumed a priori conditions on our general understanding of these relationships, Pearson correlations, and the outcome of the MLR (Appendix S2: Tables S3 and S4). Accordingly, land cover features affect hydrology, and these features together influence lake physico‐chemistry. The physical and chemical properties of lakes then interact with bathymetry and together further influence GPP_lake‐average_ and autotrophic structuring (Figure 2). The land cover type “deciduous forest” was accompanied by boreal (coniferous) forest and represented a small fraction of the land cover compared to boreal forest, and open areas and boreal forests were inversely related. Therefore, coniferous and wetland cover were included as land cover variables, whereas the main drivers of GPP_lake‐average_ and autotrophic structuring (DOC, Kd, CO_2_, *T*_water_, %*A*_littoral_, *z*_avg_) were included as water physico‐chemistry variables. We only included variables that had a path coefficient >0.3 and *p* < .05 in the path figure, although the final model includes all (also nonsignificant correlations) variables as defined in the SEM. For the parametric tests we checked all data for underlying assumptions and log‐transformed if needed (benthic, pelagic, and total GPP_lake‐average_) or for DR because it is a ratio. For two lakes, pelagic GPP_lake‐average_ was not measured, and these lakes were thus removed from the autotrophic structuring and total GPP_lake‐average_ analyses, but their water physico‐chemistry and benthic GPP_lake‐average_ values were included in the other analyses. Benthic GPP_lake‐average_ in one site (Gravatjärnen) was detected as an outlier but was retained in the analysis because this lake is an “outlier” also in a physical context by being very shallow, high in temperature, nutrients, CO_2_, and DOC, and thus we assume that the high value is a true representation.

## 3 RESULTS

### 3.1 GPP and autotrophic structuring

**FIGURE 3:**
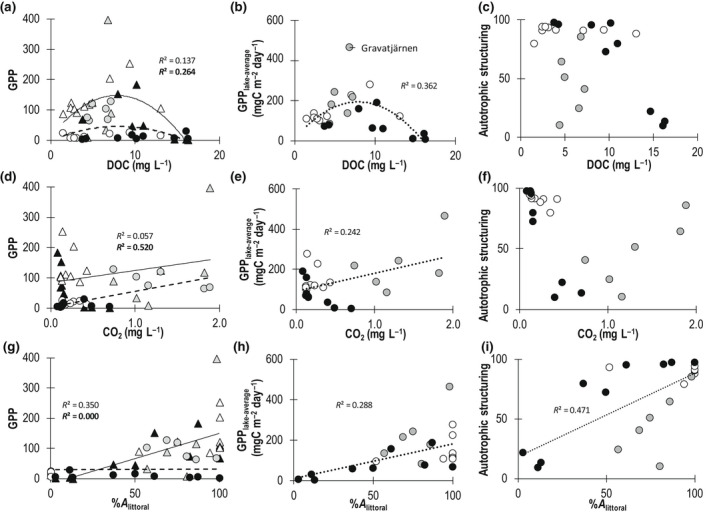
Relationships between GPP in the (a, d, g) benthic (triangles, continuous line, bold bottom *R* ^2^ values) and pelagic (circles, dashed line, top *R* ^2^ values) habitat, (b, e, h) GPP_lake‐average,_ and (c, f, i) autotrophic structuring and (a–c) DOC, (d–f) CO_2_, and (g–i) %*A*_littoral_. GPP measurements are in mg carbon (C) m^−2^ day^−1^, and autotrophic structuring represents the percent of GPP_lake‐average_ taking place in the benthic. Values indicated per biome: open = Arctic, gray = subarctic, black = boreal. DOC, dissolved organic carbon (mg L^−1^); CO_2_, carbon dioxide in lake water (mg L^−1^); %*A*_littoral_, relative areal size of the littoral benthic habitat (%)

Benthic, pelagic, and total GPP_lake‐average_ showed a unimodal relationship with DOC over the landscape (Figure 3a,b), where the autotrophic structuring was generally dominated by benthic GPP_lake‐average_ at DOC concentrations <12 mg L^−1^, except for five lakes located in the subarctic (Figure 3c). In addition, the differences in water chemistry resulted in clearly different structuring forces of GPP among the regions. Arctic lakes had generally lower DOC concentrations, and occurred on the left side of the unimodal relationship between DOC and GPP, hence GPP showed a positive response to increasing DOC concentrations. In contrast, most of the boreal lakes occurred on the right side of the unimodal relationship, where GPP decreased with increasing DOC. The subarctic lakes had higher GPP than the other biomes, with a majority occurring around the peak in GPP along the DOC gradient (Figure 3a,b).

Pelagic GPP_lake‐average_ increased with CO_2_ concentrations (52.0% of variance) over the landscape, whereas benthic GPP was unrelated to CO_2_ (Figure 3d). This relationship led to a moderate, positive correlation between total GPP_lake‐average_ and CO_2_ concentrations (24.2%). Autotrophic structuring at very low CO_2_ concentrations was benthic dominated, but at higher CO_2_ concentrations, variation in this metric increased and whole‐lake productivity became more pelagic dominated (Figure 3e–f). Yet, we observed clear differences between GPP and CO_2_ among regions: subarctic lakes had notably high CO_2_ concentrations and GPP_lake‐average_ compared to the other regions (Figure 3b,e), resulting in more variable autotrophic structuring with increased CO_2_ (Figure 3c,f). In contrast, the GPP and the autotrophic structuring declined with increased CO_2_ concentrations in the boreal lakes, likely because this also corresponded to a gradient of increasing DOC and Kd. Benthic GPP, but not pelagic GPP, was positively correlated (explaining 35.0%) with the relative amount of the littoral zone (%*A*_littoral_: relative area of sediment area receiving >1% of incoming light; Figure 3g). Thus, both the total GPP and the autotrophic structuring also increased with increasing benthic habitat (%*A*_littoral_; 28.8% and 47.1%, respectively), yet GPP was more pelagic dominated in the subarctic lakes than other lakes with similar %*A*_littoral_ (Figure 3h,i).

### 3.2 Landscape properties, bathymetry, and lake physico‐chemistry

**FIGURE 4:**
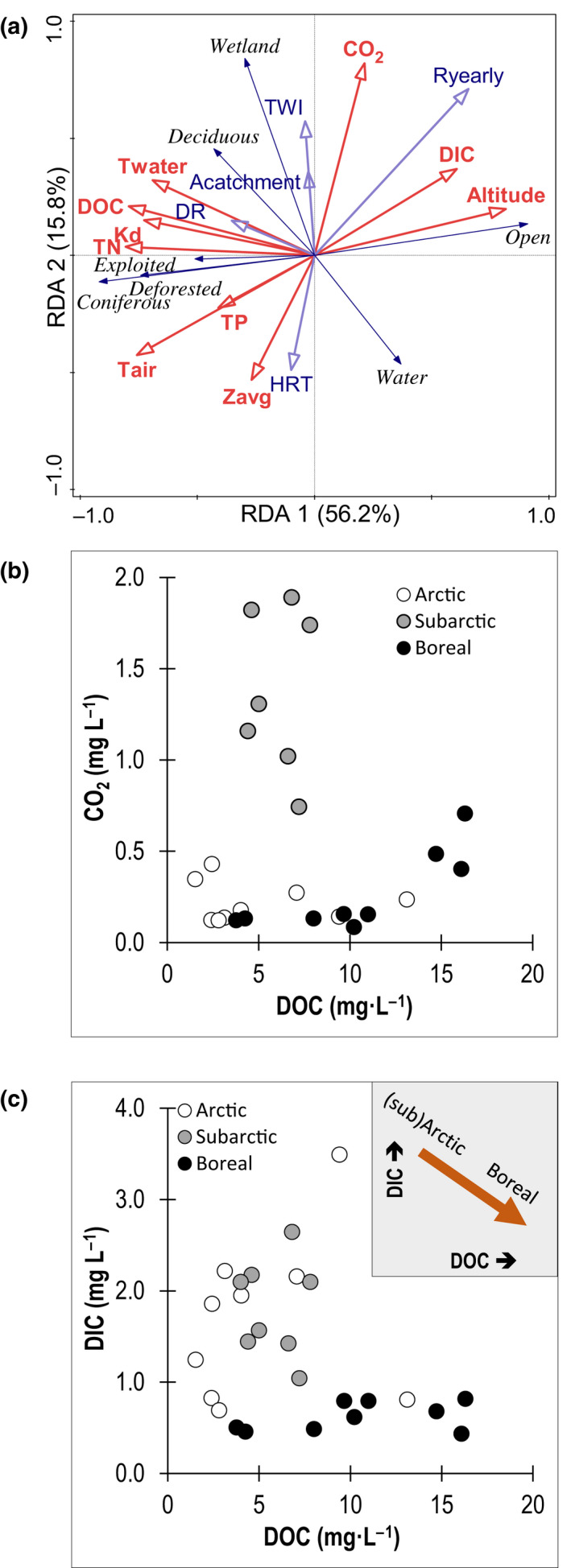
(a) Redundancy analysis showing the variation in water chemistry dataset (including the additional variables average lake depth, altitude, and monthly air temperature; red, bold font) and seven land cover variables (black, italic font), with the hydrology (purple, bold font) dataset plotted as supplementary variables. Relationship per biome between (b) CO_2_, (c) DIC, and DOC. DOC, dissolved organic carbon (mg L^−1^); DIC, dissolved inorganic carbon (mg L^−1^); CO_2_, carbon dioxide in lake water (mg L^−1^); TN, total nitrogen (mg L^−1^); TP, total phosphorus (μg L^−1^); *T*_water_, temperature at 0.2 m (°C); *T*_air_, previous monthly average air temperature (°C); *z*_avg_, average lake depth (m); *R*_yearly_, average yearly runoff (mm); TWI, topographic wetness index; DR, drainage ratio; HRT, hydraulic retention time (year^−1^), *A*_catchment_, catchment area (ha).

Land cover, together with altitude, bathymetry, and hydrology, captured a large portion of the variability in lake biogeochemical characteristics as revealed in the RDA biplot (Figure 4a; Appendix S2: Table S4 for statistics). The first RDA axis explained 56.2% of the variance in water physico‐chemistry and land cover, and captured positive associations between forest (deciduous, deforested, and coniferous) and exploited land cover and DOC concentrations. DOC clustered together with Kd, *T*_water_, TN, and to a lesser extent TP, and these variables were all negatively associated with altitude lakes and positively associated with high drainage ratios (DR). By contrast, high altitude catchments with open land cover and with higher runoff (*R*_yearly_) were linked to lakes with greater DIC concentrations. The second RDA axis explained 15.8% of the variance in lake physico‐chemistry and land cover, and mostly captured variation in wetland cover, topographic wetness (TWI), and water cover, with wetlands and TWI related to higher CO_2_ concentrations in shallow lakes (negative correlation with *z*_avg_) that tended to have shorter water residence time (HRT). Overall, DOC and CO_2_ concentrations were unrelated when considering all sites (Figure 4a). However, when assessing these relationships per biome, it becomes clear that the decoupling of CO_2_ and DOC occurs in the subarctic lakes, while CO_2_ and DOC were positively correlated when pooling the Arctic and the boreal biomes (linear regression; *R* ^2^ = .24 *p* = .04; Figure 4b). DOC and DIC concentrations on the other hand, were inversely related over this northern landscape but not within each biome (Figure 4a,c).

### 3.3 The role of landscape properties on GPP and autotrophic structuring

**FIGURE 5:**
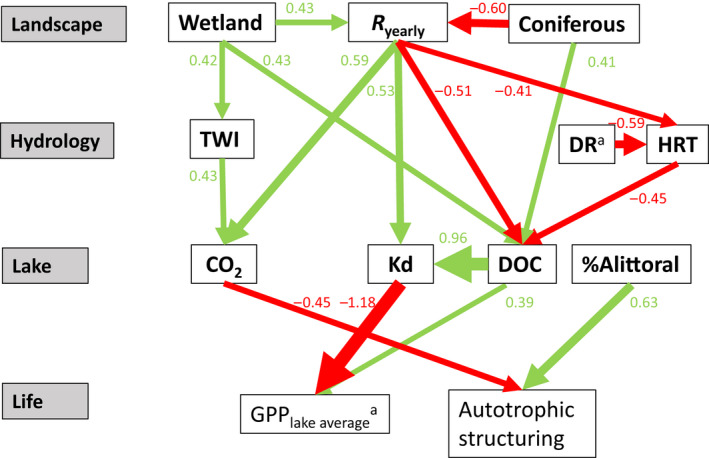
Visualization and quantification of the path analysis, depicting how landscape and hydrology variables affect lake water chemistry, and, in turn, GPP_lake‐average_ and autotrophic structuring, following the structural equation model in Figure 2. Path coefficients in red show a negative relationship and green depicts a positive relationship, and size and color of arrow corresponds to path coefficient. Variables marked with superscript “a” are log‐transformed, and only relationships between variables with a path coefficient >0.3 and *p* < .05 are displayed.

We constructed a path analysis relating total GPP_lake‐average_ and autotrophic structuring to each other, and to lake properties, which are in turn related to hydrology and catchment properties (Figure 2). The resulting model (Appendix S2: Table S6) did not meet proposed cutoff values (i.e., chi‐square *p* > .05, CFI > 0.95, TLI > 0.9, and RMSEA < 0.06) as proposed by Fan et al. (2016). Although no single index should be considered as an absolute criterion (Fan et al., 2016), we acknowledge that the outcome of our model should be interpreted carefully, and rather presents a conceptual framework for the role of landscape properties on GPP and autotrophic structuring. Autotrophic structuring increased with %*A*_littoral_ (path coefficient [PC] = 0.63) and decreased with CO_2_ concentrations (PC = −0.45), and lakes with benthic‐dominated production did not significantly affect GPP_lake‐average_ values (PC = 0.17; Figure 5; Appendix S2: Table S6). GPP_lake‐average_, on the other hand, was mostly constrained by Kd (PC = −1.18) but also increased with DOC (PC = 0.39). However, DOC had a stronger indirect negative effect on GPP_lake‐average_ through its strong influence over Kd (PC = 0.96).

DOC concentrations were negatively related to HRT (PC = −0.45), and HRT decreased with larger DR (PC = −0.59). Thus, catchments with a larger DR resulted in lower HRT and thus higher DOC concentrations (Figure 5). Lake depth (*z*_avg_) did not substantially affect GPP nor the autotrophic structuring (PC = 0.29, so just below threshold of 0.3); however, lake bathymetry is incorporated in %*A*_littoral_, a parameter that combines water clarity and bathymetry. So overall, %*A*_littoral_ and CO_2_ influenced the variation autotrophic structuring, while DOC (both directly and indirectly via Kd) affected GPP_lake‐average_. DOC, Kd, and CO_2_ were in turn linked to landscape properties; that is, increases in Kd and CO_2_, and decreases in DOC were all related to increased *R*_yearly_ (PC = 0.59, 0.53, and − 0.51, respectively). Thus, *R*_yearly_ had the potential to both enhance and inhibit GPP_lake‐average_, through increased CO_2_ concentrations (enhancement) and by increasing Kd (inhibition). Wetland cover was associated with increases in *R*_yearly_ (PC = 0.42), TWI (PC = 0.42), and DOC (PC = 0.43); increasing TWI was then associated with greater CO_2_ concentrations (PC = 0.43). By comparison, coniferous vegetation was linked to greater DOC concentrations (PC = 0.41) but lower *R*_yearly_ (PC = −0.60).

## 4 DISCUSSION

Our results show how shifts in catchment features can interact with lake bathymetry to shape broad‐scale patterns of lake productivity across Arctic, subarctic, and boreal Sweden. Specifically, regional variation in total GPP_lake‐average_ (benthic + pelagic), and in autotrophic structuring (represented by the benthic share of total GPP), reflected the dual influences of DOM concentrations on light and nutrient availability, but was also modified by the effects of bathymetry on the relative availability of benthic habitat, and was potentially stimulated by CO_2_‐fertilization. These results further imply that large‐scale global changes that affect terrestrial ecosystems and hydrological routing have the potential to reshape patterns of GPP and autotrophic structuring across northern landscapes. In addition, as in situ benthic measurements are rare, our results provide valuable empirical insights into benthic GPP, and especially autotrophic structuring in multiple northern lakes, which to our knowledge has not yet been assessed across such a steep climate gradient.

### 4.1 Drivers of GPP and autotrophic structuring

**FIGURE 6:**
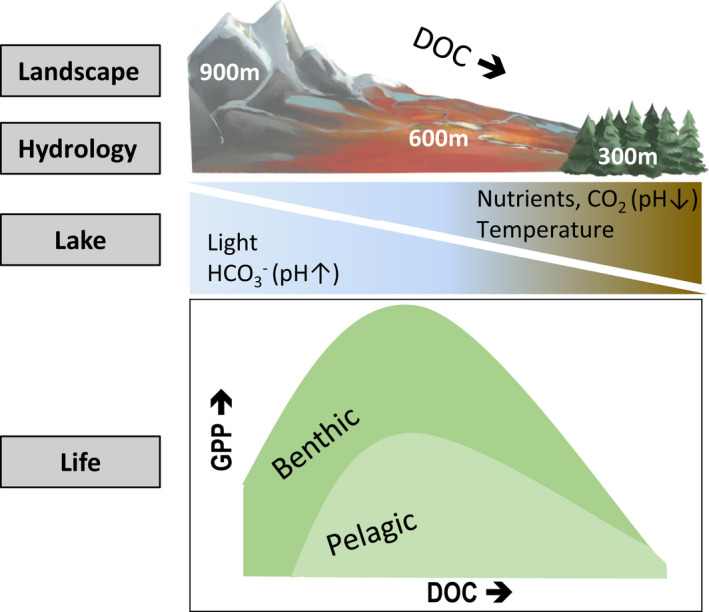
Conceptual figure of our major results, depicting a gradient (from left to right) in colored DOC driven by altitude and temperatures across the Arctic (high altitude, open areas), subarctic (mid altitude, wetlands), and boreal (low altitude, coniferous vegetation) northern landscape. Along this landscape gradient, lake water becomes browner (i.e., decreasing light), more nutrient rich, warmer, and lower in pH (shifting the dominating carbonate species in the DIC pool from ${\mathrm{HCO}}_{3}^{-}$ to CO_2_), resulting in a unimodal relationship between total GPP_lake‐average_ and DOC. The total GPP_lake‐average_ is composed of decreasing benthic (dark green) GPP, and a unimodal relationship between pelagic (light green) GPP and DOC, along the DOC gradient.

Our results are consistent with other studies on the influences of DOM on lake GPP through light limitation and nutrient supply (Hanson et al., 2003; Seekell et al., 2015; Solomon et al., 2015), which give rise to unimodal relationships between pelagic, benthic, and total GPP_lake‐average_ and DOC (the carbon fraction of DOM) concentration (Figure 3a–c). Here, GPP peaked at DOC concentrations of around 8–10 mg L^−1^ (Figure 3a,b), which is similar to thresholds found elsewhere for pelagic (Bergström & Karlsson, 2019; Solomon et al., 2015; Thrane et al., 2014), benthic (Hanson et al., 2003), and total lake GPP (Seekell et al., 2015). Similar thresholds have also been found for fish production, suggesting that this pattern is maintained through the trophic ladder (Finstad et al., 2014). Despite this similarity, there was high variability in GPP at intermediate DOC concentrations (Figure 3a,b), which is potentially related to differences in DOM properties such as nutrient availability and coloration, that alter DOC‐GPP relationships (Isles et al., 2021; Kelly et al., 2018; Seekell et al., 2015), or the direct effects of CO_2_ on GPP (Figures 3e and 6).

In contrast to GPP, autotrophic structuring across the full set of lakes was unrelated to DOC concentrations alone, but instead reflected the relative size of the benthic habitat (%*A*_littoral_), which is determined by the interactions between light climate (Kd) and lake bathymetry (maximum lake depth; *z*_max_; see also Ask et al., 2009a; Devlin et al., 2016; Vadeboncoeur et al., 2008). Across biomes, autotrophic structuring (%) was highest and most homogeneous for the Arctic lakes (average: 90%; min–max: 78%–94%), which all tended to be relatively shallow and clear. By comparison, the range for boreal (10%–97%) and subarctic (10%–85%) lakes was notably broader. Across boreal lakes, autotrophic structuring declined strongly with Kd (Pearson *r* = −.95, *p* = .01, *n* = 9), whereas the direct influence of lake geometry on littoral area (sediment area receiving >1% of incoming light) appeared to be more important among the less‐colored subarctic lakes. While these within‐biome assessments are fraught by low statistical power, our results suggest important differences in the potential for autotrophic structuring to respond to current and future environmental changes. For example, browning and associated increases in Kd in boreal lakes will likely have strong, negative impacts on autotrophic structuring, whereas for shallow and clear Arctic lakes, increases in water color would need to be dramatic to shade benthic production and alter autotrophic structuring. For subarctic lakes, autotrophic structuring could shift from being controlled by bathymetry, to being controlled by Kd with increased lake browning.

Among subarctic lakes, the positive correlation between CO_2_ and GPP suggests that carbon fertilization may act as an additional driver of productivity and autotrophic structuring (Figure 3e,f). Specifically, autotrophic structuring increased with CO_2_ concentrations among the subarctic lakes (*r* = .76, *p* < .05, *n* = 6), consistent with some degree of benthic carbon fertilization, whereas this same metric declined with CO_2_ across boreal lakes (*r* = −.92, *p* < .01, *n* = 9), and did not change with variation in CO_2_ across Arctic lakes (see Figure 3f). As above, these within‐biome tests should be interpreted with caution, but the opposing patterns nonetheless contrast with results from the full path analysis, which suggested a single negative relationship between CO_2_ and autotrophic structuring (Figure 5). Further, observations from subarctic lakes are consistent with studies showing that CO_2_ can promote the sum of benthic and pelagic GPP (Hamdan et al., 2018). Similarly, co‐limitation by CO_2_ and nutrients on pelagic GPP has been demonstrated in experimental and field studies in oligotrophic northern lakes (Jansson et al., 2012). The potential mechanisms behind CO_2_ fertilization, or co‐fertilization of CO_2_ and nutrients, could include that enzymes active in photosynthesis are not fully saturated at CO_2_ levels close to equilibrium, and/or that the carbon‐concentrating mechanisms of algal cells are downregulated under nutrient‐limited conditions (see Jansson et al., 2012 and references therein). Yet, significant changes (positive or negative) in autotrophic structuring due to limiting access to CO_2_ are only expected in lakes where there is sufficient light throughout the water column to promote either benthic or pelagic GPP (i.e., on the left side of the hump; Figure 3a,b). Thus, lakes with high concentrations of colored DOC (i.e., on the right side of the hump, Figure 3a,b) are likely resistant to such effects, as light limitation operates as an overwhelming control over GPP (Figure 3a,b,f). In addition, considering the dominance of benthic processes in many northern shallow lakes (Karlsson & Säwström, 2009), it would take considerable increases in pelagic GPP in response to CO_2_ and/or nutrients (Bergström & Karlsson, 2019; Vadeboncoeur et al., 2003) to *decrease* autotrophic structuring. Indeed, while experimental studies are needed to test these mechanisms, results from the subarctic lakes suggest that increasing the supply of CO_2_ may instead *increase* autotrophic structuring through stimulating effects on benthic GPP.

### 4.2 Links between lakes and landscapes

The environmental drivers of GPP_lake‐average_ and autotrophic structuring across lakes reflect the influence of catchment properties, but also how these influences are modified locally by hydrology (Figures 5 and 6; Bergström, 2020). For example, higher DOC concentrations and increased color occurred in lakes with more extensive wetland and coniferous forest cover in the surrounding catchment, but were also elevated in lakes with high drainage ratios (DRs) and short hydrological water retention times (HRTs; Figure 5), in line with other studies (Kothawala et al., 2014). Hence, for boreal lakes, scenarios of catchment greening, increased precipitation and recovery of acidification (De Wit et al., 2016; Finstad et al., 2016; Monteith et al., 2007) that can lead to greater DOC mobilization are likely to reduce total GPP and autotrophic structuring if lake DOC concentrations are near the threshold where light limitation emerges (Figure 3b). On the other hand, catchments with open land cover and high yearly runoff (*R*_yearly_), conditions mostly found at subarctic and high altitude Arctic sites, were associated with lower DOC concentrations (Figures 4a and 5), likely caused by low terrestrial inputs (Jansson et al., 2008) and dilution (Isles et al., 2018; Larsen, Andersen, et al., 2011). Lake browning in these biomes is only likely to occur when the effects of catchment greening (leading to higher DOC) are not offset by increases in precipitation (leading to lower DOC). Under this scenario, enhanced DOC concentrations likely promote total GPP with a benthic driven autotrophic structuring in Arctic lakes (see Figure 3a,d), whereas subarctic lakes are potentially driven towards the right side of the unimodal distribution with risk of lowered total GPP due to light limitation. The direction of the autotrophic structuring is less predictable since it relates to changes in CO_2_ concentrations. In this context, CO_2_ concentrations in lakes also increased with wetland cover and wet soils (e.g., Wallin et al., 2010), but was unrelated to forest cover and increased with yearly runoff. Collectively, these patterns reinforce the influence of landscape properties on the carbon biogeochemistry of northern lakes, but also highlight the potential for internal lake processes to mediate carbon chemistry, and also suggest that different sets of landscape drivers operate for DOC versus CO_2_.

One result of these distinct landscape controls is that the major biogeochemical drivers of GPP and autotrophic structuring, DOC and CO_2_, were not related when assessing all lakes combined (Figure 3b) and were distinct in the subarctic (Figure 3b). In fact, DOC and CO_2_ were only positively correlated when considering boreal lakes alone (*R* ^2^ = .65, *p* = .01) or when combining boreal and Arctic lakes (*R* ^2^ = .24, *p* = .04), supporting previous studies from this region (Arctic: Jonsson et al., 2003; boreal: Sobek et al., 2003) and elsewhere (Rantakari & Kortelainen, 2008; Roehm et al., 2009). This relationship is thought to arise from microbial oxidation of DOC in the water column and/or sediments, which generates CO_2_ (Jonsson et al., 2001, 2003; Larsen, Andresen, et al., 2011). However, our data also indicate that CO_2_–DOC relationships can vary among regions (Rantakari & Kortelainen, 2008; Weyhenmeyer et al., 2015). Specifically, subarctic lakes had higher CO_2_ than both Arctic and boreal counterparts, likely due to a combination of a large DIC pool combined with relatively low pH (Figure 4b and see Section 3), whereas DIC concentrations were relatively high and similar in subarctic and Arctic lakes, but lower in the boreal lakes (Figure 4c). Overall, this uncoupling points to important external inputs of inorganic carbon to Arctic and subarctic lakes, which could be linked to deep groundwater sources (e.g., respiration) from surrounding soils (Striegl & Michmerhuizen, 1998), but more likely reflect greater inputs of geogenic DIC from mineral weathering (Campeau et al., 2017; Humborg et al., 2010). Resolving the importance of internal versus multiple external carbon sources is further complicated by variation in HRT, which adds noise to DOC–CO_2_ relationships, depending how these different pools are altered over time within lakes (e.g., through DOC processing and CO_2_ outgassing; Vachon et al., 2020).

Given the influence of both DOC and CO_2_ on lake productivity, understanding how these carbon inputs will change as high latitude landscapes warm is a priority. For example, in subarctic and Arctic environments, inorganic carbon releases have been linked to thawing of permafrost in wetland and tundra systems with thick organic soils (peatlands; Dabrowski et al., 2020; Dimova & Burnett, 2011; Weyhenmeyer et al., 2015). However, our Arctic and subarctic study regions are characterized by sporadic to isolated permafrost (Åkerman & Johansson, 2008; Gisnås et al., 2017; Appendix S2: Figure S3), suggesting lower impacts from permafrost thaw on carbon supply to lakes when compared to other landscapes at comparable latitudes, for example, in North America and Siberia (e.g., Kuhn et al., 2021; Vonk et al., 2015 and references therein). Regardless, other ecosystem changes in northern Fennoscandia may alter carbon inputs and fate in lakes as the climate warms, including increases in terrestrial productivity (Finstad et al., 2016), and changes in hydrology (De Wit et al., 2016). The amount of bioavailable inorganic carbon (CO_2_) for primary producers relative to the DIC pool is determined by pH, which additionally varies over the landscape. Our results imply that any changes in land‐use, climate and hydrology, acid recovery and potentially permafrost thawing have the potential to impact catchment production, release, and delivery of DOC, DIC, and CO_2_. These drivers together impact lake light (and nutrient) conditions, carbonate systems, and pH, and have the potential of influencing GPP and autotrophic structuring of northern lakes (Figure 6).

## 5 CONCLUSION

The regional patterns of production reported here have important implications for northern lakes. The overall primary production, as well as the relative contribution of pelagic versus benthic production in these ecosystems can have a cascading set of consequences for higher trophic levels, impacting the productivity and community composition of zooplankton, benthic invertebrates, and fish (Karlsson et al., 2015; Kelly et al., 2014; van Dorst et al., 2019). The balance between nutrients and light as well as autotrophic community composition will also affect elemental ratios and essential biochemical compounds for consumers (Müller‐Navarra, 2008; Peltomaa et al., 2017; Sterner & Hessen, 1994). Specifically, carbon fertilization in the pelagic (Brown et al., 2019; Grasset et al., 2020; Jansson et al., 2012) may increase the carbon to nutrient ratios in algae, and thus affect the nutritional quality for zooplankton (Hessen et al., 2002; Urabe et al., 2002). Benthic autotrophs, both in terms of benthic algae and vascular plants often support high densities of consumers (Devlin et al., 2013; Karlsson & Säwström, 2009; Liboriussen & Jeppesen, 2006; Norman et al., 2022) both by providing shelter for benthic animals and younger fish but also by providing a high‐energy food source. Thus, lakes with benthic driven primary production may reflect more optimal feeding opportunities for consumers within lakes (Karlsson & Byström, 2005). The interactive effects of high DOC (and thus low light), temperature, and pelagic versus benthic production may also influence the productivity of fish and induce shifts between dominant salmonids in these systems like brown trout and Arctic char (Finstad et al., 2011). Our results show that catchment‐derived organic and inorganic carbon are dominant factors for structuring GPP across northern lakes. Colored DOC is the strongest determinant of the total lake GPP, whereas the autotrophic structuring is shaped by the relative size of the benthic habitat (%*A*_littoral_, i.e., indirectly by DOC via light shading and directly via bathymetry) and may be modified by carbon fertilization in low DOC and low pH lakes. Our results underline that the impacts of global change on the productivity of northern lakes will be mediated by the interactions between landscape structure, hydrology, and lake bathymetry, which together determine the total productivity and the structuring of GPP into the benthic versus pelagic habitats. Our results aid in predicting global change impacts on GPP and autotrophic structuring, moderating habitat structuring and resource quantity for aquatic consumers.

## CONFLICT OF INTEREST

Authors declare no conflict of interest.

## Supporting information


Appendix S1

Appendix S2
Table S1
Table S2
Table S3
Table S4a
Table S4b
Table S5
Table S6
Table S7
Figure S1
Figure S2
Figure S3

## Data Availability

The data that support the findings of this study are openly available at https://doi.org/10.5061/dryad.vx0k6djvs.
