# Supplementary material for: Landscape determinants of pelagic and benthic primary production in northern lakes
Source: Glob Chang Biol. 2022 Sep 15;28(23):7063–77. doi: 10.1111/gcb.16409 (PMC9826228; doi:10.1111/gcb.16409)
Supplement: Supplementary file 1 — Appendix S1 [file GCB-28-7063-s001.docx]

**Supporting information**. Puts et al., 2022. Landscape determinants of pelagic and benthic primary production in northern lakes.

# Appendix S1 on method specification

## **Water physico-chemistry and bathymetry**

Samples for pH, DOC, DIC, total N (TN) and P (TP) were taken from the epilimnion (i.e. at 1m depth) where the water is well mixed, and where most of the pelagic GPP occurs, or were taken from composite water samples in unstratified lakes (nine of the lakes from Ask et al. (2009)). Water for DOC was filtered through a 0.45µm filter (Sarstedt Filtropur), acidified using HCl to an end concentration of 12mM, and stored in a refrigerator before analyses. DOC concentrations in the water from the subarctic sites was analyzed through high temperature combustion in a Formacs HT-I analyzer (Skalar), and water from the boreal and Arctic sites by the high temperature catalytic oxidation method (HTCO) using a Shimadzu TOC-5000 (Jenck Instrumental S.A., Buenos Aires, Argentine Republic). Total nitrogen (TN) and phosphorus (TP) (unfiltered) samples were stored frozen, until analysis on a segmented flow analyzer (QuAAtro 39, Seal Analytical; method no. Q-115-10 Rev. 4) for water from the subarctic lakes. For water from the Arctic and boreal lakes, TP was analyzed according to Murphy and Riley (1962) after oxidative hydrolysis with potassium peroxodisulphate, and TN was obtained after analyzing Kjeldahl- N (Jönsson, 1966) and nitrate plus nitrite (Grasshoff et al., 1983). Dissolved inorganic carbon (DIC) samples from the subarctic sites were taken by injecting 4mL (air free) water into a tightly sealed 18mL glass vial (pre-flushed with N_2_) containing 0.1mL 1.2M HCl. Water for the DIC samples from the boreal and Arctic sites were collected from 1 m depth, gently transferred to 118-mL glass bottles (n = 3) and immediately acidified with 1 mL 1.2 mol L^-1^ HCl after the bottles had been sealed air-tight. In the laboratory, a 10mL He headspace was created using two syringes with attached thin needles as temporary sampling ports, and the bottles were shaken for one minute. The concentration of CO_2_ in the headspace (vials and bottles) was analyzed as soon as possible on a Clarus 500 gas chromatograph (Perkin Elmer) using N_2_ as carrier gas for water from the subarctic samples, and on a CP4900 (Varian Inc., Palo Alto, California, USA) gas chromatograph for the other sites. The DIC concentrations in the lake water were then calculated using the headspace CO_2_ concentration, water temperature, water and headspace volumes, and air pressure according to Åberg et al. (2007). pH was measured immediately after sampling in the laboratory, and CO_2_ concentrations in the lake water were calculated from DIC, pH and temperature, following guidelines from the Water Quality Analysis Simulation Program by the United States Environmental Protection Agency ([https://www.epa.gov](https://www.epa.gov/sites/default/files/2018-05/documents/wasp-ph-release-notes.pdf) for specifics).

## **Gross primary production (GPP)**

**Benthic GPP: DIC & Dome method**

Benthic GPP was measured using the “DIC-method” in the Arctic and boreal biome, and using the “Dome-method” in the subarctic, with either triplicate measurements at the shallowest depth (DIC-method) or seasonal measurements representing replicates (Dome-method). For the DIC-method, intact sediment cores with overlaying water were incubated for about 24h at the depth of collection, and GPP was measured by tracking changes in DIC over the incubation period in sealed off dark (respiration) and transparent (respiration (R) + GPP) incubation tubes. For each tube, pelagic GPP and respiration in the overlaying water were subtracted, and values (mg C L^-1^) were related to the water volume and the benthic area in the incubation tubes and the precise time of incubation (Ask et al., 2009). For the Dome-method, three transparent half-sphered dome equipped with a miniDOT oxygen logger (recording date, time, O_2_, and temperature with an interval of 1 or 5 minutes; MiniDOT website) were softly placed on the sediment (at three different depths per lake) covering the available sediment area for about 24 hours. The domes had a total incubation area of 0.091m^2^, inner volume of 14.8L, and were attached to a metal frame of 0.05m that penetrated the sediment to seal off the dome from outside O_2_. For each dome, benthic GPP (mg O_2_ L^-1^ day^-1^) was derived from net ecosystem production (NEP) and by assuming that GPP equals NEP plus respiration (R). NEP, GPP and R were calculated using the R-package Lake Metabolizer (Winslow et al., 2016) with the 'metab.bookkeep´ function based on the metabolism model of Cole et al. (2000), by setting the air-gas exchange parameter ´k´ to 0, and by removing negative GPP values from the dataset (see Puts et al. (2022) for specifics, discussion, and sensitivity). This model assumes oxygen dynamics to be the result of NEP (GPP+R) during daytime and R during nighttime. For each dome, GPP in oxygen units (mg O_2_ L-^1^ day^-1^) were converted into carbon (mg C L^-1^ day^-1^), assuming CO_2_ consumption and O_2_ generation were aligned, hence, using a photosynthetic quotient of 1 (Bender et al., 1987). We neglected pelagic processes in the water above the sediment in the Dome-method because of a lower pelagic volume to benthic area relationship compared to in the tubes in the DIC-method. Benthic GPP for each dome or core (mg C·m^-2^ ·day^-1^) was calculated by relating the GPP in mg C L^-1^ day^-1^ to the incubation area and volume, and the actual time of incubation.

**Pelagic GPP**

We measured pelagic GPP *in situ* at the surface and at subsequent 1m depth intervals, with additional measurements at 0.25 and 0.5m, where the deepest measurement depended on the lake depth and water color. Measurements were done by incubating transparent glass bottles filled with water from the sampling depth, with additional incubations in dark bottles at the most shallow and deepest measurements, for about four hours midday at the same depth the samples were taken from, using ^14^C isotopic tracer as described by (Schindler, Schmidt, & Reid, 1972). We took replicate measurements at the most shallow and deepest measurements, and we verified that DIC did not become limiting during the 4h incubation by estimating the consumption of the DIC pool in the incubation bottles, showing that less than 1% of the total DIC pool was consumed. Calculated hourly GPP values were extrapolated to daily GPP using the ratio of incident photosynthetically active radiation (PAR) during incubation in relation to daily PAR.

**Lake averages of benthic and pelagic GPP**

The GPP rates measured at discrete depths within a lake were upscaled to a single average GPP estimate per lake, for both the benthic and pelagic GPP, and used throughout this paper. Lake-averages of benthic GPP (mg C m^-2^ day^-1^) were calculated by integrating the GPP rates from 2-5 depths over the corresponding lake surface area per depth interval and relating the sum to the total lake area (See figure 1 below). Lake surface area is slightly smaller than the sediment bottom area, implying that we may slightly underestimate benthic GPP. Lake-averages of pelagic GPP were calculated by integrating the depth-specific rates over the water column and dividing them by the lake surface area using volume-weighted depth-specific pelagic GPP rates when upscaling to a lake average (Appendix S2; Figure 1, see below).


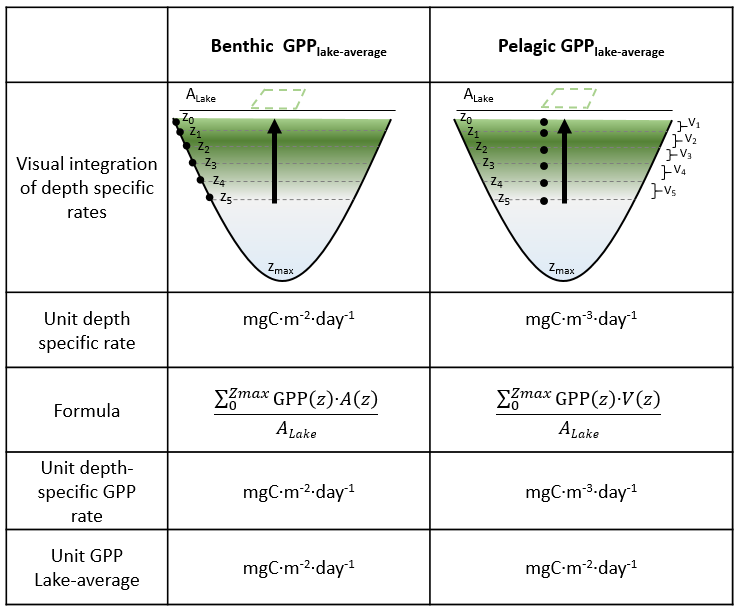


Figure S1: Graphical and mathematical visualization of the upscaling of benthic and pelagic GPP rates measured at discrete depths (black dots), and the units of rates and lake-averages. Although here we represent five depths, the amount of sample points varied per lake with 3-5 for the benthic, and up to 10 for pelagic GPP.

## **References:**

Åberg, J., Jansson, M., Karlsson, J., Nääs, K.-J., & Jonsson, A. (2007). Pelagic and benthic net production of dissolved inorganic carbon in an unproductive subarctic lake. *Freshwater Biology*, *52*(3), 549–560. https://doi.org/https://doi.org/10.1111/j.1365-2427.2007.01725.x

Ask, J., Karlsson, J., Persson, L., Ask, P., Byström, P., & Jansson, M. (2009). Whole-lake estimates of carbon flux through algae and bacteria in benthic and pelagic habitats of clear-water lakes. *Ecology*, *90*(7), 1923–1932. https://doi.org/10.1890/07-1855.1

Bender, M., Grande, K., Johnson, K., Marra, J., Williams, P. J. L. B., Sieburth, J., … Heinemann, K. (1987). A comparison of four methods for determining planktonic community production. *Limnology and Oceanography*. https://doi.org/10.4319/lo.1987.32.5.1085

Cole, J. J., Pace, M. L., Carpenter, S. R., & Kitchell, J. F. (2000). Persistence of net heterotrophy in lakes during nutrient addition and food web manipulations. *Limnology and Oceanography*. https://doi.org/10.4319/lo.2000.45.8.1718

Grasshoff Ehrhardt, Manfred., Kremling, Klaus., K. (1983). *Methods of seawater analysis*. Weinheim; Deerfield Beach: Verlag chemie.

Jönsson, E., Vattnehygien (Water Hygiene, in Swedish), 1966, 22, 10. NB: Water samples conserved with HgCl2.

MiniDOT website. (n.d.). Retrieved July 30, 2022, from https://www.pme.com/new-products/minidot-usb-oxygen-logger.

Murphy J, Riley JP. 1962. A modified single-solution method for the determination of phosphate in natural waters. Anal Chim Acta 27:31–36

Puts, I. C., Bergström, A. ‐K., Verheijen, H. A., Norman, S., & Ask, J. (2022). An ecological and methodological assessment of benthic gross primary production in northern lakes. Ecosphere, 13(3), 1–16. <https://doi.org/10.1002/ecs2.3973>

Schindler, D. W., Schmidt, R. V., & Reid, R. A. (1972). Acidification and Bubbling as an Alternative to Filtration in Determining Phytoplankton Production by the 14 C Method . Journal of the Fisheries Research Board of Canada, 29(11), 1627–1631. https://doi.org/10.1139/f72-250

Winslow, L. A., Zwart, J. A., Batt, R. D., Dugan, H. A., Iestyn Woolway, R., Corman, J. R., … Read, J. S. (2016). LakeMetabolizer: An R package for estimating lake metabolism from free-water oxygen using diverse statistical models. *Inland Waters*, *6*(4), 622–636. https://doi.org/10.5268/IW-6.4.883
