# Supplementary material for: Landscape determinants of pelagic and benthic primary production in northern lakes
Source: Glob Chang Biol. 2022 Sep 15;28(23):7063–77. doi: 10.1111/gcb.16409 (PMC9826228; doi:10.1111/gcb.16409)
Supplement: Supplementary file 2 — Appendix S2 Table S1 Table S2 Table S3 Table S4a Table S4b Table S5 Table S6 Table S7 Figure S1 Figure S2 Figure S3 [file GCB-28-7063-s002.docx]

Supporting information. Puts et al., 2022. Landscape determinants of pelagic and benthic primary production in northern lakes.

Appendix S2

Background data.

Tables;

S1: Overview parameters

S2: Land cover categories

S3: Multiple linear regression MLR

S3: Pearson correlations

S4: RDA stats

S5: Path stats

Figures:

S1: Catchments and landcover

S2: GPP & nutrients, zavg

S3: Location lakes and permafrost cover

References

*Table S1: Variables/parameters with their symbols and definition, and units.*

| **Variable/parameter** | **Symbol** | | **Source/equation** | **Unit** |
| --- | --- | --- | --- | --- |
| Depth | z | Direct measurement | | m |
| Maximum depth | z_max_ | Bathymetry | | m |
| Average lake depth | z_avg_ | V_lake_$\cdot$A_lake_^-1^ | | m |
| Lake surface area | A_lake_ | Bathymetry | | hectare |
| Euphotic depth | z_euph_ | -Ln (0.01) · Kd | | m |
| Lake volume | V_lake_ | Bathymetry | | m^3^ |
| Lake area in littoral zone | A_littoral_ | Bathymetry & z_euph_ | | m^2^ |
| Relative areal size of the littoral benthic habitat | %A_littoral_ | 100· A_littoral_$\cdot$A_lake_^-1^ | | % |
| Water temperature  (at 0.2m depth) | T_water_ | Direct measurement | | °C |
| Volume/Area between depths | V/A(z) | $\int_{z1}^{z2} V/A\left( z \right)\cdot dz$ | | m^3^/m^2^ |
| Benthic GPP rate at discrete depth | GPP*_z_* | Direct measurement | | mgC·m^-3^·day^-1^ |
| Pelagic GPP rate at discrete depth | GPP*_z_* | Direct measurement | | mgC·m^-2^·day^-1^ |
| Benthic GPP_lake-average_ | - | $\frac{\int_{0}^{Zmax} GPP\left( z \right)\text{·}A\left( z \right)\text{·}dz}{ALake}$ | | mgC·m^-2^·day^-1^ |
| Pelagic GPP_lake-average_ | - | $\frac{\int_{0}^{zmax} GPP\left( z \right)\text{·}V\left( z \right)\text{·}dz}{ALake}$ | | mgC·m^-2^·day^-1^ |
| Total GPP_lake-average_ | - | Benthic GPP_lake-average_ + Pelagic GPP_lake-average_ | | mgC·m^-2^·day^-1^ |
| Autotrophic structuring | - | $100\cdot\frac{Benthic GPPlake average}{Total GPPlake average}$ | | mgC·m^-2^·day^-1^ |
| Catchment area | A_catchment_ | See methods | | hectare |
| Drainage ratio | DR | $\frac{A\mathrm{catchment}}{A\mathrm{lake}}$ | | - |
| Topographic wetness index | TWI | See methods | | - |
| Average yearly runoff | R_yearly_ | See methods | | mm |
| Hydraulic retention time | HRT | $\frac{V\mathrm{lake}}{Annual discharge}$ | | Average yearly^-1^ |

*Table S2: Land cover assigned categories present in the catchments, using original pixel values from the Swedish “Marktäcke” (land cover) data.*

| Pixel value | Description | English description | Assigned category |
| --- | --- | --- | --- |
| 2 | Våtmark | Wetland | Wetland |
| 41 | Övrig, öppen mark utan vegetation | Other, open land without vegetation | Open |
| 42 | Övrig, öppen mark med vegetation | Other, open land with vegetation | Open |
| 51 | Exploterad mark, väg/järnvag | Exploited land, road/railway | Exploited |
| 52 | Exploterad mark, byggnad eller väg/järnvag | Exploited land, building, or road/railway | Exploited |
| 61 | Sjö/vattendrag | Lake/water body | Water |
| 111 | Tallskog utanför våtmark | Pine forest bordering wetlands | Coniferous |
| 112 | Granskog utanför våtmark | Spruce forest bordering wetlands | Coniferous |
| 113 | Barrblandskog utanför våtmark | Coniferous mixed forest bordering wetlands | Coniferous |
| 114 | Lövblandad barrskog utanför våtmark | Mixed deciduous coniferous forest bordering wetlands | Deciduous |
| 115 | Triviallövskog utanför våtmark | Unspecified deciduous forest bordering wetlands | Deciduous |
| 118 | Temporärt ej skog utanför våtmark | Temporarily deforested bordering wetlands | Deforested |
| 121 | Tallskog på våtmark | Pine forest bordering wetlands | Coniferous |
| 122 | Granskog på våtmark | Spruce forest on wetlands | Coniferous |
| 123 | Barrblandskog på våtmark | Coniferous forest on wetlands | Coniferous |
| 124 | Lövblandad barrskog på våtmark | Mixed deciduous coniferous forest on wetlands | Deciduous |
| 125 | Triviallövskog på våtmark | Unspecified deciduous forest on wetlands | Deciduous |
| 128 | Temporärt ej skog på våtmark | Temporarily deforested on wetlands | Deforested |

*Table S3. Multiple linear regressions with forward selection of four different measures of GPP. The criteria for adding predictors is based on the first highest R² value, and stop adding if the p-value of the predictor is smaller than 0.05. Additionally assessed AIC to judge the model quality and if predictors added to the models*^[[1]](#footnote-1)^*.*

| # | **Coefficients of explanatory variables** | **Intercept** | **R^2^** | **Df2** | **F-value** | **AIC** | **Deselected variables** |
| --- | --- | --- | --- | --- | --- | --- | --- |
|  | **I) Pelagic GPP_lake-average_^a^** |  |  |  |  |  |  |
| a | 0.691·CO_2_ | 0.845 | 0.505 | 20 | 20.371 | -39.27 | Altitude, DOC, TN, TP, Kd, T_water_, Area, z_avg_, z_max_, %A_littoral_, DIC, PAR |
| b | 0.584·CO_2_ -  0.123·z_avg_ | 1.332 | 0.630 | 19 | 16.15 | -43.67 | Altitude, DOC, TN, TP, Kd, T_water_, Area, z_max_, %A_littoral_, DIC, PAR |
|  | **II) Benthic GPP_lake-average_^a^** |  |  |  |  |  |  |
| a | -0.575·Kd | 2.461 | 0.729 | 20 | 53.85 | -42.70 | Altitude, DOC, CO_2_, TN, TP, T_water_, Area, z_avg_, z_max_, %A_littoral_, DIC, PAR |
| b | -0.595·Kd -0.064·z_max_ | 3.032 | 0.809 | 19 | 40.32 | -46.42 | Altitude, DOC, CO_2_, TN, TP, T_water_, Area, z_avg_, %A_littoral_, DIC, PAR |
|  | **III) Total GPP_lake-average_^a^** |  |  |  |  |  |  |
| a | -0.361·Kd | 2.453 | 0.641 | 20 | 35.65 | -52.11 | Altitude, DOC, CO_2_, TN, TP, Twater, Area, z_avg_, z_max_, %A_littoral_, DIC, PAR |
| b | -0.329·Kd -0.128·z_avg_ | 2.861 | 0.831 | 19 | 46.85 | -66.77 | Altitude, DOC, CO_2_, TN, TP, Twater, Area, z_max_, %A_littoral_, DIC, PAR |
|  | **IV) Autotrophic structuring** |  |  |  |  |  |  |
| a | -20.862·Kd | 95.215 | 0.458 | 20 | 16.88 | 142.81 | Altitude, DOC, CO_2_, TN, TP , T_water_, Area, z_avg_, z_max_, %A_littoral_, DIC |
| b | -25.695·Kd -  19.093·CO_2_ | 106.147 | 0.603 | 19 | 14.44 | 137.94 | Altitude, DOC, TN, TP, T_water_, Area, z_avg_, z_max_, %A_littoral_, DIC |

*Table S4a: Path statistics; path-coefficients and their z and p value, and if the variable is included in the visualization of the path-analysis. Abbreviations are as follows: R_yearly_= average yearly runoff (mm), DR= Drainage ratio, TWI= Topographic wetness index, HRT= Hydraulic retention time (year^-1^), DOC= dissolved organic carbon (mg·L^-1^), z_avg_= average lake depth (m), %A_littoral_ = relative areal size of the littoral benthic habitat. ^a^ denotes log transformation of the variable.*

|  | **Estimate** | **Std.Err** | **z-value** | **P(>\|z\|)** | **Std.lv** | **Std.all** | **Included?** |
| --- | --- | --- | --- | --- | --- | --- | --- |
| R_yearly_ | ~ |  |  |  |  |  |  |
| wetland | 0.007 | 0.002 | 3.648 | 0.00 | 0.007 | 0.427 |  |
| coniferous | -0.007 | 0.001 | -5.098 | 0.00 | -0.007 | -0.597 |  |
| DR^a^ | ~ |  |  |  |  |  |  |
| R_yearly_ | -0.268 | 0.316 | -0.848 | 0.40 | -0.268 | -0.17 | INSIGNIFICANT |
| TWI | ~ |  |  |  |  |  |  |
| wetland | 0.024 | 0.012 | 1.9 | 0.06 | 0.024 | 0.415 |  |
| R_yearly_ | 0.585 | 0.711 | 0.824 | 0.41 | 0.585 | 0.18 | INSIGNIFICANT |
| HRT | ~ |  |  |  |  |  |  |
| R_yearly_ | -1.072 | 0.415 | -2.584 | 0.01 | -1.072 | -0.405 |  |
| DR^a^ | -0.988 | 0.264 | -3.741 | 0.00 | -0.988 | -0.587 |  |
| T_water_ | ~ |  |  |  |  |  |  |
| DOC | 0.214 | 0.155 | 1.381 | 0.17 | 0.214 | 0.269 | INSIGNIFICANT |
| z_avg_ | -0.554 | 0.419 | -1.32 | 0.19 | -0.554 | -0.255 | INSIGNIFICANT |
| %A_littoral_ | -0.034 | 0.023 | -1.487 | 0.14 | -0.034 | -0.310 | INSIGNIFICANT |
| DOC | ~ |  |  |  |  |  |  |
| HRT | -2.235 | 0.57 | -3.925 | 0.00 | -2.235 | -0.445 | INSIGNIFICANT |
| coniferous | 0.061 | 0.025 | 2.435 | 0.02 | 0.061 | 0.413 |  |
| R_yearly_ | -9.222 | 2.706 | -3.408 | 0.00 | -9.222 | -0.693 |  |
| wetland | 0.101 | 0.036 | 2.773 | 0.01 | 0.101 | 0.434 |  |
| TWI | 0.491 | 0.527 | 0.93 | 0.35 | 0.491 | 0.120 | INSIGNIFICANT |
| z_avg_ | -0.329 | 0.34 | -0.968 | 0.33 | -0.329 | -0.120 |  |
| Kd | ~ |  |  |  |  |  |  |
| HRT | -0.045 | 0.108 | -0.421 | 0.67 | -0.045 | -0.037 | INSIGNIFICANT |
| coniferous | 0.009 | 0.004 | 2.148 | 0.03 | 0.009 | 0.245 |  |
| R_yearly_ | 1.735 | 0.487 | 3.561 | 0.00 | 1.735 | 0.533 |  |
| wetland | -0.006 | 0.006 | -0.988 | 0.32 | -0.006 | -0.107 | INSIGNIFICANT |
| TWI | -0.06 | 0.079 | -0.751 | 0.45 | -0.06 | -0.059 | INSIGNIFICANT |
| DOC | 0.235 | 0.03 | 7.778 | 0.00 | 0.235 | 0.959 |  |
| z_avg_ | 0.193 | 0.051 | 3.764 | 0.00 | 0.193 | 0.288 |  |
| CO_2_ | ~ |  |  |  |  |  |  |
| HRT | -0.155 | 0.064 | -2.442 | 0.02 | -0.155 | -0.239 | INSIGNIFICANT |
| wetland | 0.004 | 0.004 | 1.023 | 0.31 | 0.004 | 0.124 | INSIGNIFICANT |
| R_yearly_ | 1.014 | 0.287 | 3.532 | 0.00 | 1.014 | 0.589 |  |
| coniferous | 0.002 | 0.002 | 0.921 | 0.36 | 0.002 | 0.118 | INSIGNIFICANT |
| TWI | 0.225 | 0.047 | 4.818 | 0.00 | 0.225 | 0.425 |  |
| DOC | 0.016 | 0.018 | 0.889 | 0.37 | 0.016 | 0.123 | INSIGNIFICANT |
| z_avg_ | 0.033 | 0.031 | 1.069 | 0.29 | 0.033 | 0.092 | INSIGNIFICANT |
| T_water_ | 0 | 0.013 | 0.031 | 0.98 | 0 | 0.002 | INSIGNIFICANT |
| GPP_lake-average_^a^ | ~ |  |  |  |  |  |  |
| %A_littoral_ | -0.002 | 0.001 | -1.257 | 0.21 | -0.002 | -0.092 | INSIGNIFICANT |
| Kd | -0.571 | 0.073 | -7.78 | 0.00 | -0.571 | -1.179 |  |
| CO_2_ | 0.235 | 0.063 | 3.719 | 0.00 | 0.235 | 0.257 |  |
| T_water_ | 0.013 | 0.01 | 1.233 | 0.22 | 0.013 | 0.085 | INSIGNIFICANT |
| DOC | 0.046 | 0.017 | 2.711 | 0.01 | 0.046 | 0.390 |  |
| z_avg_ | -0.083 | 0.026 | -3.19 | 0.00 | -0.083 | -0.257 |  |
| Autotrophic Structuring | ~ |  |  |  |  |  |  |
| %A_littoral_ | 0.598 | 0.14 | 4.268 | 0.00 | 0.598 | 0.626 |  |
| Kd | -7.612 | 8.498 | -0.896 | 0.37 | -7.612 | -0.272 | INSIGNIFICANT |
| CO_2_ | -23.657 | 7.32 | -3.232 | 0.00 | -23.657 | -0.447 |  |
| T_water_ | 1.115 | 1.184 | 0.942 | 0.35 | 1.115 | 0.129 | INSIGNIFICANT |
| DOC | 0.462 | 1.978 | 0.233 | 0.82 | 0.462 | 0.067 | INSIGNIFICANT |
| zavg | 1.347 | 3.023 | 0.446 | 0.66 | 1.347 | 0.072 | INSIGNIFICANT |
| GPP_lake-average_^a^ | ~ |  |  |  |  |  |  |
| Autotrophic Structuring | 0.003 | 0.002 | 1.8 | 0.07 | 0.003 | 0.172 | INSIGNIFICANT |

*Table S4b:Outcome lavaan model and test runs of the path analysis.*

| lavaan 0.6-9 ended normally after 206 iterations |
| --- |
|  |
| Estimator ML |
| Optimization method NLMINB |
| Number of model parameters 54 |
|  |
| Number of observations 24 |
|  |
| Model Test User Model: |
|  |
| Test statistic 143.776 |
| Degrees of freedom 41 |
| P-value (Chi-square) 0.000 |
|  |
| Model Test Baseline Model: |
|  |
| Test statistic 420.654 |
| Degrees of freedom 85 |
| P-value 0.000 |
|  |
| User Model versus Baseline Model: |
|  |
| Comparative Fit Index (CFI) 0.694 |
| Tucker-Lewis Index (TLI) 0.365 |
|  |
| Loglikelihood and Information Criteria: |
|  |
| Loglikelihood user model (H0) -268.855 |
| Loglikelihood unrestricted model (H1) -196.967 |
|  |
| Akaike (AIC) 645.710 |
| Bayesian (BIC) 709.325 |
| Sample-size adjusted Bayesian (BIC) 542.033 |
|  |
| Root Mean Square Error of Approximation: |
|  |
| RMSEA 0.323 |
| 90 Percent confidence interval - lower 0.267 |
| 90 Percent confidence interval - upper 0.381 |
| P-value RMSEA <= 0.05 0.000 |
|  |
| Standardized Root Mean Square Residual: |
|  |
| SRMR 0.153 |
|  |
| Parameter Estimates: |
|  |
| Standard errors Standard |
| Information Expected |
| Information saturated (h1) model Structured |

*Table S5: RDA statistics.*

| **Water chemistry and land cover (Fig 4a)** | | | | | | |
| --- | --- | --- | --- | --- | --- | --- |
| Statistic | Axis 1 | Axis 2 | Axis 3 | Axis 4 | Cases | 26 catchments, incl. 3 supplementary |
| Eigenvalues | 0.562 | 0.158 | 0.054 | 0.027 | Response variables | 7 land cover units |
| Explained variation (cumulative) | 56.24 | 72.08 | 77.45 | 80.16 | Expl. vars | 10 environmental variables |
| Pseudo-canonical correlation | 0.948 | 0.877 | 0.873 | 0.739 | Suppl.vars | 5 hydrological variables |
| Explained fitted variation (cumulative) | 68.35 | 87.60 | 94.13 | 97.42 |  |  |
| Pseudo-canonical correlation (suppl.) | 0.812 | 0.812 | 0.647 | 0.550 |  |  |

*Table S6: Pearson correlations between pelagic GPP_lake average_ (in mg C·m^-2^·day^-1^), physio chemical, bathymetry, and seven land cover variables. Abbreviations are as follows: GPP= GPP_lake-average_, AutStr= autotrophic structuring, Alt= Altitude, DOC= dissolved organic carbon (mg·L^-1^), DIC= dissolved inorganic carbon (mg·L^-1^), CO_2_= carbon dioxide in lake water (mg·L^-1^), TN= total nitrogen (mg·L^-1^), TP= total phosphorus (µg·L^-1^), PAR= daily incoming PAR at surface (W·m^-2^), T_water_ = temperature at 0.2m (°C), T_air_ = previous monthly average air temperature (°C), Area= lake surface area (m^2^), z_avg_= average lake depth (m), A_catch._= catchment area (ha), rest is described in Appendix S1 Table S1 & S2. ^a^ denotes log transformation of the variable, and all correlations displayed have a p<0.05.*

|  | GPP^a^ | AutStr | Alt | DOC | DIC | CO_2_ | TN | TP | PAR | Kd | %A_littoral_ | T_water_ | T_air_ | Area | z_avg_ | A_catch._ | R_yearly_ | DR^a^ | TWI | HRT | water | wetland | open | coniferous | deciduous | deforested | exploited |
| --- | --- | --- | --- | --- | --- | --- | --- | --- | --- | --- | --- | --- | --- | --- | --- | --- | --- | --- | --- | --- | --- | --- | --- | --- | --- | --- | --- |
| GPP^a^ | 1 | .54 |  | -.57 | .51 |  | -.50 | -.70 | -.41 | -.78 | .76 |  | -.49 |  | -.55 |  | .49 |  |  |  |  |  |  | -.56 |  |  |  |
| AutStr | .54 | 1 |  | -.49 |  | -.45 |  |  |  | -.68 | .69 |  |  |  |  |  |  | -.49 |  |  |  |  |  |  |  |  |  |
| Alt. |  |  | 1 |  | .62 |  |  |  |  |  |  |  | -.59 |  | -.44 |  |  |  |  |  |  |  | .46 | -.54 |  | -.46 | -.49 |
| DOC | -.57 | -.49 |  | 1 |  |  | .90 | .73 | .46 | .88 | -.69 | .48 | .46 | -.49 |  |  | -.47 | .67 |  |  | -.52 |  | -.69 | .58 | .46 | .53 |  |
| DIC | .46 |  | .42 |  | 1 | .42 |  |  |  | -.42 |  |  | -.44 |  | -.41 |  |  |  |  |  |  |  | .63 | -.54 |  |  |  |
| CO_2_ |  | -.52 |  |  | .42 | 1 |  |  | -.60 |  |  |  |  |  |  |  | .73 |  | .70 |  |  | .73 |  |  |  |  |  |
| TN | -.50 |  |  | .90 |  |  | 1 | .75 | .46 | .72 | -.51 | .54 | .54 | -.43 |  | .40 | -.65 | .67 |  |  |  |  | -.68 | .66 | .39 | .51 |  |
| TP | -.70 |  |  | .73 |  |  | .75 | 1 | .57 | .70 | -.57 |  |  |  |  |  | -.59 | .61 |  |  |  |  |  |  |  |  |  |
| PAR | -.41 |  |  | .46 | -.42 | -.65 | .46 | .57 | 1 |  |  |  |  |  |  |  | -.75 |  | -.48 |  |  | -.49 |  | .47 |  | .53 |  |
| Kd | -.78 | -.68 |  | .88 | -.40 |  | .72 | .70 |  | 1 | -.85 | .39 | .48 | -.40 |  |  |  | .59 |  |  | -.47 |  | -.63 | .58 |  | .42 |  |
| %A_littoral_ | .76 | .69 |  | -.69 |  |  | -.51 | -.57 |  | -.85 | 1 | -.40 | -.45 |  |  |  |  | -.50 |  |  |  |  | .54 | .54 |  |  |  |
| T_water_ |  |  |  | .48 |  |  | .54 |  |  | .39 | -.40 | 1 | .61 |  |  |  |  |  |  |  |  |  | -.71 | .48 | .44 | .43 | .48 |
| T_air_ | -.49 |  | -.59 | .46 | -.59 |  | .54 |  |  | .48 | -.45 | .61 | 1 | -.57 |  |  | -.59 |  |  |  |  |  | -.69 | .72 |  | .47 | .49 |
| Area |  |  |  | -.49 |  |  | -.43 |  |  | -.40 |  |  | -.57 | 1 |  |  |  |  |  |  |  |  |  |  |  |  |  |
| z_avg_ | -.55 |  | -.44 |  | -.41 |  |  |  |  |  |  |  |  |  | 1 |  |  |  |  | .52 | .56 |  |  | .40 |  |  |  |
| A_catch._ |  |  |  |  | .43 |  | .40 |  |  |  |  |  |  |  |  | 1 |  | .72 |  |  |  |  |  |  |  |  |  |
| R_yearly_ | .49 |  |  | -.47 | .48 | .79 | -.65 | -.59 | -.75 |  |  |  | -.59 |  |  |  | 1 |  | .43 |  |  | .61 | .45 | -.72 |  | -.53 |  |
| DR^a^ |  | -.49 |  | .67 |  |  | .67 | .61 |  | .59 | -.50 |  |  |  |  | .72 |  | 1 |  | -.52 |  |  |  |  |  | .42 |  |
| TWI |  |  |  |  |  | .72 |  |  | -.48 |  |  |  |  |  |  |  | .43 |  | 1 |  |  | .59 |  |  |  |  |  |
| HRT |  |  |  |  |  |  |  |  |  |  |  |  |  |  | .52 |  |  | -.52 |  | 1 | .79 |  |  |  |  |  |  |
| Water |  |  |  | -.52 |  |  |  |  |  | -.47 |  |  |  |  | .56 |  |  |  |  | .79 | 1 |  |  |  | -.47 |  |  |
| Wetland |  |  |  |  |  | .79 |  |  | -.49 |  |  |  |  |  |  |  | .61 |  | .59 |  |  | 1 |  |  |  |  |  |
| Open |  |  | .46 | -.69 | .51 |  | -.68 |  |  | -.63 | .54 | -.71 | -.69 |  |  |  | .45 |  |  |  |  |  | 1 | -.80 |  | -.54 | -.48 |
| Coniferous | -.56 |  | -.54 | .58 | -.62 |  | .66 |  | .47 | .58 | .54 | .48 | .72 |  | .40 |  | -.72 |  |  |  |  |  | -.80 | 1 |  | .52 | .48 |
| Deciduous |  |  |  | .46 |  |  | .39 |  |  |  |  | .44 |  |  |  |  |  |  |  |  | -.47 |  |  |  | 1 |  |  |
| Deforested |  |  | -.46 | .53 |  |  | .51 |  | .53 | .42 |  | .43 | .47 |  |  |  | -.53 | .42 |  |  |  |  | -.54 | .52 |  | 1 |  |
| Exploited |  |  | -.49 |  |  |  |  |  |  |  |  | .48 | .49 |  |  |  |  |  |  |  |  |  | -.48 | .48 |  |  | 1 |

*Table S7: RDA statistics.*

| **Water chemistry and land cover (Fig 4a)** | | | | | | |
| --- | --- | --- | --- | --- | --- | --- |
| Statistic | Axis 1 | Axis 2 | Axis 3 | Axis 4 | Cases | 26 catchments, incl. 3 supplementary |
| Eigenvalues | 0.562 | 0.158 | 0.054 | 0.027 | Response variables | 7 land cover units |
| Explained variation (cumulative) | 56.24 | 72.08 | 77.45 | 80.16 | Expl. vars | 10 environmental variables |
| Pseudo-canonical correlation | 0.948 | 0.877 | 0.873 | 0.739 | Suppl.vars | 5 hydrological variables |
| Explained fitted variation (cumulative) | 68.35 | 87.60 | 94.13 | 97.42 |  |  |
| Pseudo-canonical correlation (suppl.) | 0.812 | 0.812 | 0.647 | 0.550 |  |  |

*Figure S1 : Catchments*
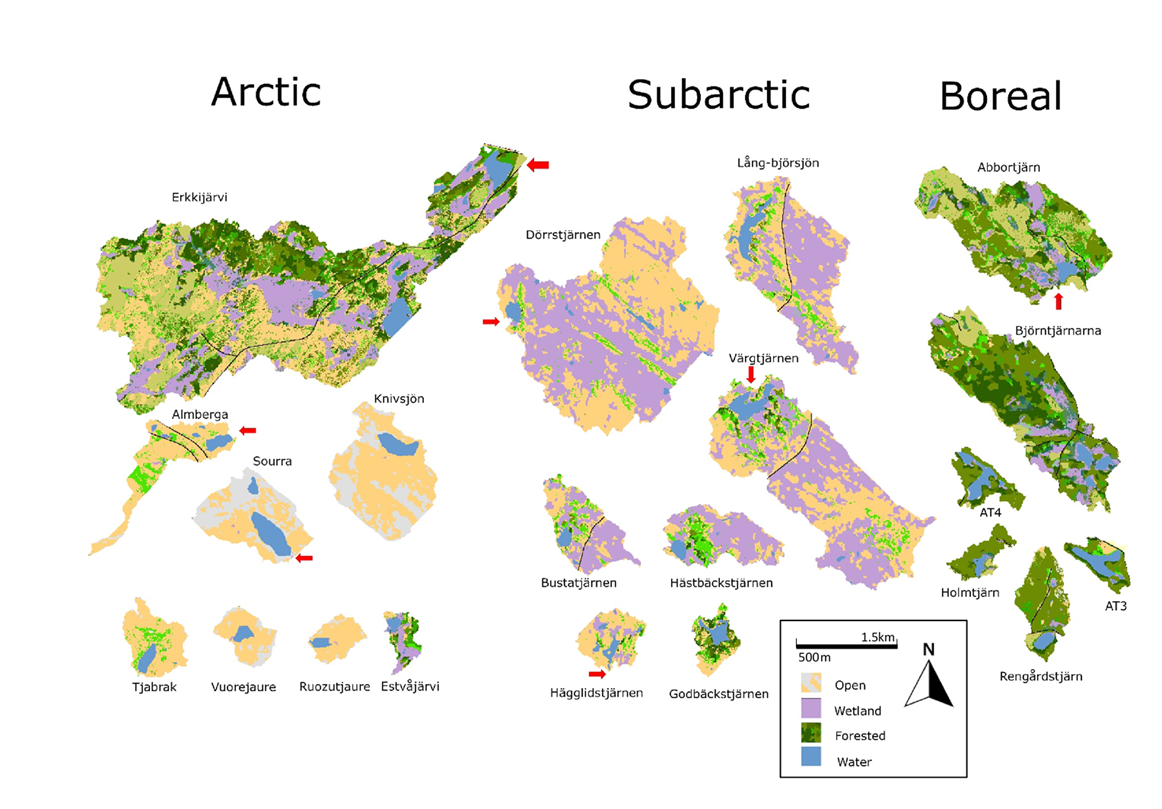


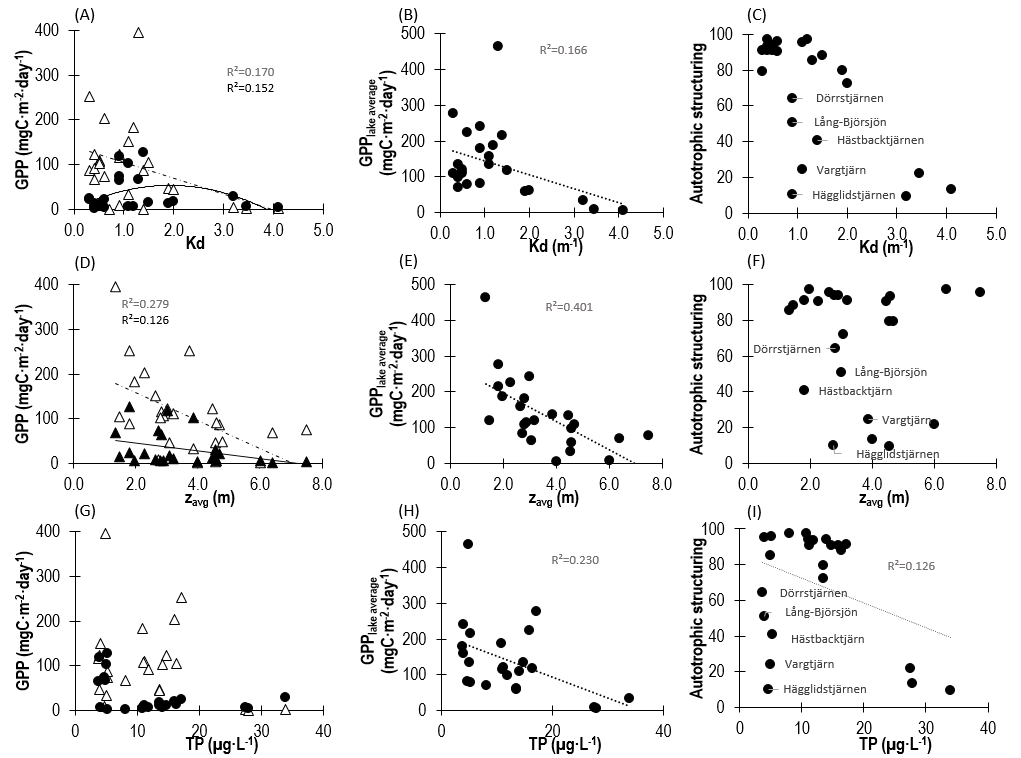


*Figure S2: Relationships between (a-c) Kd, (d-f) z_avg_ and (g-i) TP, and GPP in the (a,d,g) benthic (open triangles, dark grey R^2^ values) and pelagic (black triangles and R^2^ values) habitat, (b,e,h) GPP_lake-average_ and (c,f,i) autotrophic structuring. GPP measurements are in mg carbon (C) m^-2^ day^-1^, and autotrophic structuring represents the percent of GPP_lake-average_ taking place in the benthic. Abbreviations; z_avg_= average lake depth (m^-1^), TP = total phosphorus (µg L^-1^).*


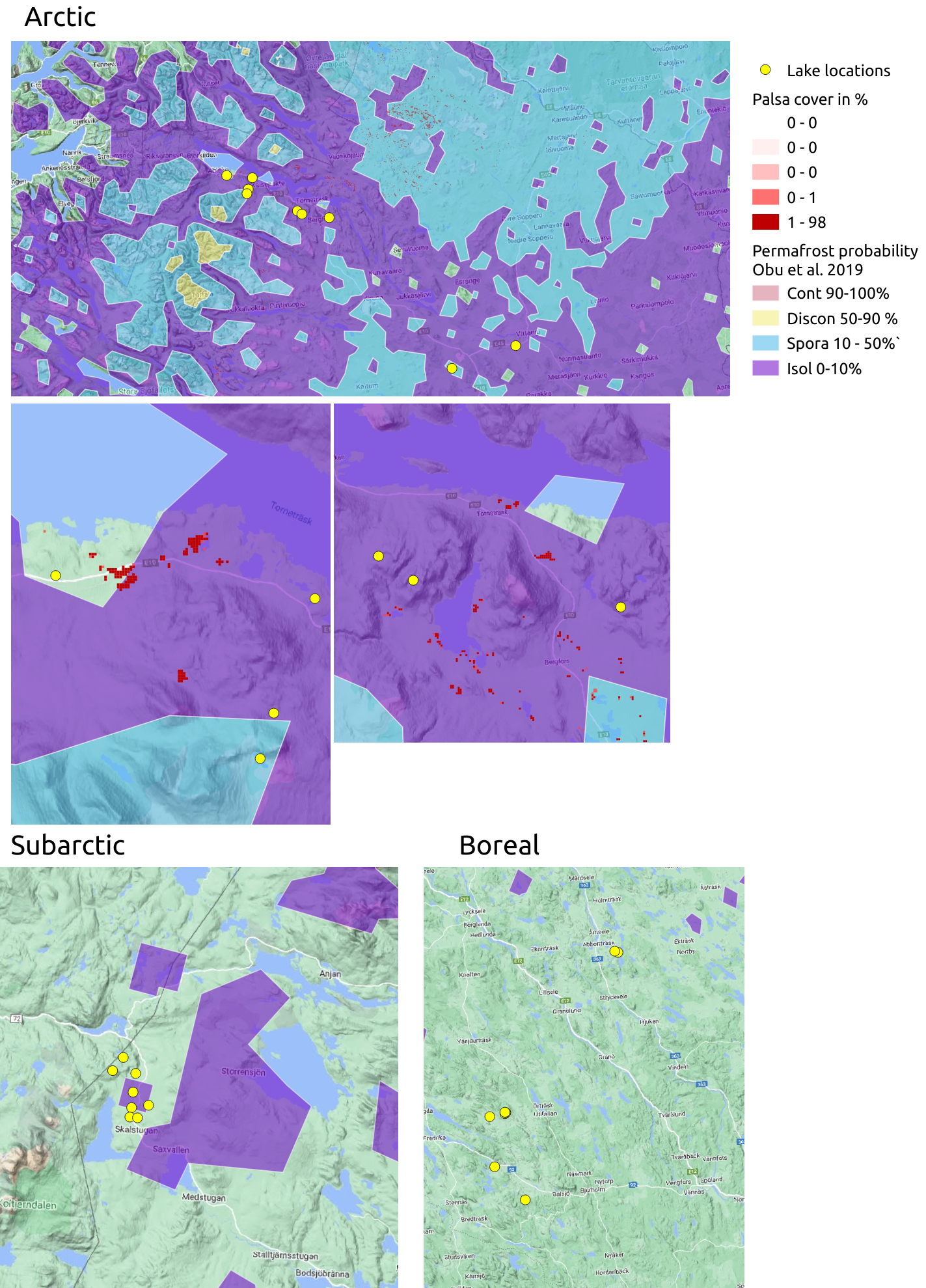


*Figure S3: Modeled permafrost distribution and the location of our study lakes sensu* Obu et al., (2019) *and the distribution of palsas mapped for ~2010 together with the lake locations.*

**References**

Obu, J., Westermann, S., Bartsch, A., Berdnikov, N., Christiansen, H. H., Dashtseren, A., … Zou, D. (2019). Northern Hemisphere permafrost map based on TTOP modelling for 2000–2016 at 1 km2 scale. *Earth-Science Reviews*, *193*(October 2018), 299–316. https://doi.org/10.1016/j.earscirev.2019.04.023

1. *Note: Variables marked with ”^a^” are log-transformed. All models are significant at the p<0.05 level. Abbreviations: df2, degrees of freedom (denominator); AIC, Akaike’s information criterion; DOC, dissolved organic carbon (mg L^-1^); TN, total nitrogen (µg L^-1^); TP, total phosphorus (µg L^-1^), Kd, light attenuation coefficient of the water (m^-1^); T_water_, water temperature at 0.2m depth (in °C); Area, surface area of the lake (m^2^); z_avg_, average lake depth (m); ; z_max_, maximum lake depth (m); %A_littoral_, relative areal size of the littoral benthic habitat (%); DIC, dissolved inorganic carbon (mg L^-1^); PAR, daily incoming photosynthetic active radiation (in Watt m^-2^).* [↑](#footnote-ref-1)
